# Psoriasis *de novo* or exacerbation by PD-1 checkpoint inhibitors^☆^

**DOI:** 10.1016/j.abd.2023.09.003

**Published:** 2024-02-21

**Authors:** Zi Wan, Jiangyuan Huang, Xiaojie Ou, Shuang Lou, Jianji Wan, Zhu Shen

**Affiliations:** a The Second School of Clinical Medicine, Southern Medical University, Guangzhou, China; b Department of Dermatology, Guangdong Provincial People's Hospital (Guangdong Academy of Medical Sciences), Southern Medical University, Guangzhou, China

**Keywords:** Immune checkpoint inhibitors, Immunotherapy, Psoriasis

## Abstract

PD-1 (programmed Death-1) immune checkpoint inhibitors have provided significant benefits to tumor patients. However, a considerable proportion of the patients develop immune-related adverse events (irAEs), of which cutaneous irAEs (cirAEs, e.g., psoriasis) occur relatively early. This review provides an overview of the current progress in psoriasis *de novo* or exacerbation by PD-1 checkpoint inhibitors. It not only describes the relevant influencing factors but also theoretically analyzes the immunological mechanisms that lead to the onset or exacerbation of psoriasis. Finally, the authors present guidelines for the treatment of psoriasis *de novo* or exacerbation by PD-1 checkpoint inhibitors. The review is intended to assist dermatologists in the early recognition and effective individualized management of such cirAE, which is helpful to continue or adjust the tumor-targeted immunotherapy on the basis of ensuring the quality of life of tumor patients.

## Introduction

PD-1 (Programmed Death-1) checkpoint inhibitors are widely used in oncology immunotherapy and have provided significant clinical benefits to oncology patients. However, more than 50% of the patients develop immune-related adverse events (irAEs), of which cutaneous irAEs (cirAEs) occur relatively early. Psoriatic skin lesions, as one of the cirAEs, not only affect patients' quality of life but may also require adjustment of PD-1 inhibitor regimens due to the severity of the skin lesions. In this review, the authors focus on the current research on psoriasis *de novo* or exacerbation by PD-1 checkpoint inhibitors, including clinical manifestations, prediction and diagnosis, possible mechanisms, and treatment principles. The aim is to improve the recognition and effective individualized management of these cirAEs by dermatologists to ensure the quality of life for cancer patients.

## Checkpoint PD-1 in the maintenance of T-cell homeostasis

It is now clear that T-cell activation is tightly regulated by a “dual channel” to avoid under- or over-reaction. The first signal refers to the specific binding of the T-Cell Receptor (TCR) to the Major Histocompatibility Complex (MHC) of the Antigen-Presenting Cells (APCs). The second signal refers to the interactions of immune checkpoint molecules (co-stimulatory and co-inhibitory molecules) on the surface of the T-cells and APCs. Co-inhibitory immune checkpoints are a class of immunosuppressive molecules that include programmed death receptors and their ligands. They can prevent sustained immune responses, thus avoiding damage and destruction of normal tissues. Common co-suppressive immune checkpoints include PD-1, CTLA-4 (Cytotoxic T-Lymphocyte-Associated Protein 4), VISTA (V-domain Ig Suppressor of T-cell Activation), TIM (CD366; HAVCR2) and LAG-3 (Lymphocyte Activation Gene 3).

PD-1 is a co-inhibitory immune checkpoint that is widely targeted in clinical therapy. The human PD-1 gene is located on chromosome 2q37.3. It is a transmembrane protein molecule consisting of 288 amino acid residues. It belongs to the immunoglobulin superfamily.^1^ As a regulatory receptor, it is mainly expressed on activated CD4+ and CD8+ T-cells. Unactivated T-cells do not express high-level PD-1. PD-1 is also expressed on B cells, NKT cells, dendritic cells and monocytes.^2^ The PD-1 molecule binds mainly to its ligand PD-L1 (B7-H1) or PD-L2 (B7-DC). PD-L1 and PD-L2 can be expressed in tumor cells, dendritic cells, macrophages, and vascular endothelial cells.

The binding of PD-1 to the ligand inhibits the TCR signaling pathway mediated by the tyrosine phosphatase SHP2. By providing negative costimulatory signals, it inhibits T-cell activation and proliferation, leading to reduced T-cell function, loss of activity, and/or apoptosis. It can be seen that PD-1 plays an important role in the establishment of peripheral immune tolerance.^3^, ^4^

## PD-1 checkpoint inhibitors and their irAEs in tumor targeting therapy

Tumor cells express PD-L1 and thus exploit the normal physiological checkpoints of immune cells for immunosuppression. This can lead to an imbalance between tumor growth and host surveillance, so anti-PD-1 drugs may reverse the inhibitory signal from tumor cells to immune cells, particularly CD8+ T-cells. In 2010, scientists reported the first study of anti-PD-1 antibodies in solid tumors, including 39 patients with advanced melanoma, non-small cell lung cancer, renal cell carcinoma, prostate cancer and colorectal cancer treated with MDX1106 (nivolumab).^5^ Currently, nivolumab and pembrolizumab are the most widely used anti-PD-1 antibodies in clinical practice. They have shown significant therapeutic efficacy in a wide range of tumors, including advanced melanoma, non-small cell lung cancer, renal cell carcinoma, bladder cancer, and Hodgkin's lymphoma.^6^

Immune-related adverse events may occur in the targeted therapy of tumors, known as irAEs. irAEs have the following characteristics: (i) Organ specificity: irAEs can affect almost any organ system in the body, but the most commonly affected tissues are the body's barrier tissues, such as the skin, gastrointestinal tract, liver and respiratory epithelium; (ii) Their delayed onset and long-term persistence: although the mean time to onset of irAEs varies among individuals, they all have the characteristic of delayed onset, typically occurring weeks even months after drug administration;^7^ (iii) Inflammatory memory background: patients with a concomitant or previous inflammatory disease are more likely to develop irAEs; (iv) Reversibility: 70% of irAEs are mild to moderate and most resolve after discontinuation of the drug or use of corticosteroids;^8^, ^9^, ^10^ (v) Tumor type heterogeneity: the same immune checkpoint inhibitors (ICIs) may produce different toxicity profiles when used to treat different tumors; (vi) ICI-related incidence: anti-CTLA-4 results in a higher overall incidence of irAEs than anti-PD-l. The incidence of irAEs increased when the combination was administered.

Common irAEs include dermal toxicity, endocrine toxicity, hepatotoxicity and immune-related pneumonia, being skin-related the most common irAEs; on the other hand, cardiac and nephrotoxicity are rare irAEs events.^11^, ^12^, ^13^, ^14^ The incidence of cutaneous toxicity due to anti-CTLA-4 and anti-PD-1 is 50% and 30%‒40%, respectively.^15^ Cutaneous irAEs may manifest as non-specific rash, psoriasis, vitiligo, lichenoid dermatitis, pemphigus, aspergillosis, dermatomyositis, and severe drug rash.

## The characteristics of psoriasis *de novo* or exacerbation by PD-1 checkpoint inhibitors

PD-1-induced psoriasis can be either *de novo* (55.0%) or an exacerbation of pre-existing psoriasis. The most common clinical type is plaque psoriasis (70.6%), with guttate psoriasis (21.4%) and rare pustular and reverse psoriasis manifestations.^16^, ^17^, ^18^ Furthermore, the involvement of palmoplantar areas can be found in 38.3% of PD-1-induced psoriasis.^19^

Psoriasis, as a cirAE caused by PD-1 inhibitor, currently lacks effective prediction tools, and it is difficult to achieve model prediction for its occurrence in tumor patients who use ICIs. However, integrating factors related to the disease may be helpful for its diagnosis: (i) Concomitant psoriasis or history/family history of psoriasis; (ii) Psoriatic lesions appearing or exacerbation on average 5‒12 weeks or longer after the start of ICI therapy;^20^, ^21^ (iii) Matching the distribution and lesion characteristics of classic psoriatic lesions;^22^, ^23^, ^24^ (iv) The histopathological features of PD-1 inhibitor-related psoriasis are not significantly different from those of classic psoriasis. However, drug-induced psoriasis usually lacks significantly dilated dermal papillary capillaries and thinning of the papillary upper epidermis;^25^, ^26^ (v) Psoriasis treatment protocol is effective for the lesions.

Studies have shown that those tumor patients who developed irAE had better outcomes than those who did not develop irAE.^27^, ^28^ In a study with 7008 tumor patients, those with concomitant psoriasis had better survival than patients without psoriasis (HR = 0.703; 95% Confidence Interval 0.497‒0.994; p = 0.045).^29^ The development of psoriasis, a complication of PD-1 inhibitors, may be a reflection of a good response to tumor PD-1 inhibitor treatment. However, this conclusion needs to be supported by studies with larger sample sizes.

## The theoretical mechanism of psoriasis *de novo* or exacerbation by PD-1 checkpoint inhibitors

Since PD-1 inhibitors can reduce or release the “brakes” on the immune response, they can theoretically activate the innate and adaptive immune systems. The cells that may be affected by PD-1 inhibitors include dendritic cells, Th cells, and Treg cells. The cytokines they secrete, especially interferon-gamma, IL-1, IL-17, and IL-22, may play a vital role in the pathogenesis of psoriasis *de novo* or exacerbation.^30^ However, the exact mechanisms need to be verified by further studies. Here the authors present a step-by-step theoretical analysis of the potential effects of PD-1 inhibitors on the innate and adaptive immune systems.

### Effects of PD-1 inhibitors on the innate immune system

#### Potential effects of PD-1 inhibitors on neutrophils

As a key driver of the innate immune response, neutrophils play an important role in the axis of psoriasis pathogenesis. Neutrophils shuttle out of the blood vessels and migrate into the epidermis to form the Munro or Kogoj microabscess, the typical pathological feature of psoriasis.^31^ Currently, it is believed that neutrophils secrete various cytokines and chemokines such as TNF-α, IL-17, and IL-36 family factors, as well as neutrophil extracellular traps (NETs) and neutrophil exosomes, directly or indirectly involved in the pathogenesis of psoriasis.

The PD-L1/PD-1 pathway is involved in the regulation of neutrophil apoptosis. PD-L1 on neutrophils contains a structure similar to tyrosine-X-X-Methionine (YXXM), which promotes the binding of Phosphatidylinositol 3-Kinase (PI3K) and activates the p85 subunit. The latter regulates apoptosis of the neutrophils themselves, thereby prolonging their activity.^32^, ^33^ Thus, anti-PD-1 antibodies may prolong neutrophil activity and are likely to participate in the onset and maintenance of psoriasis through the aforementioned pathways.

#### Potential effects of PD-1 inhibitors on dendritic cells

There are three types of dendritic cells (DCs) in psoriasis lesions: langerhans cells (LCs), plasmacytoid dendritic cells (pDCs) and myeloid dendritic cells (mDCs). The former are mainly found in the epidermis of psoriatic lesions, while the latter two are mainly found in the dermis, and all three play a pathogenic role in psoriasis.^34^

At present, there is no investigation to elucidate the mechanism of DCs in psoriasis induced by PD-1 inhibitors, but the authors can obtain clues from research in the field of oncology. It has been shown that PD-L1 can be expressed on tumor cells and Tumor-Infiltrating Immune Cells (TICs), particularly macrophages and myeloid DCs. PD-1/PD-L1 signal in tumor microenvironment can mediate the inhibition of T-cells.^35^, ^36^ PD-1 inhibitors have been demonstrated to directly induce Interferon γ (IFN-γ) production of activated T-cells, which induces IL-12 production by intra-tumor DC subpopulations. IL-12 is involved in both anti-tumor T-cell immunity and stimulates massive T-cell proliferation while secreting various cytokines to form a positive feedback inflammatory loop. In addition, IL-12 can inhibit *Eomes*, a key regulator of T-cell exhaustion, resulting in reduced T-cell exhaustion.^37^ Similarly, the authors know that IL-12 is also one of the factors that play an important role in the onset and development of psoriasis.^38^ However, research is needed to confirm whether these mechanisms are related to the involvement of DCs in the induction or exacerbation of psoriasis by PD-1 inhibitors.

#### Potential effects of PD-1 inhibitors on macrophages

Macrophages, as one of the key cells of innate immunity, have the role of producing inflammatory mediators, presenting antigens, phagocytosis, and killing pathogenic microorganisms. According to their immune function, they can be divided into M1-type macrophages, i.e. classically activated macrophages, and M2-type macrophages, i.e. alternatively activated macrophages.^39^ In terms of immune function, M1-type macrophages and M2-type macrophages represent two extremes. M1 activation mainly secretes some pro-inflammatory factors that help Th1 cells in their anti-infective function. In contrast, M2 activation is mainly involved in fibrosis, tissue repair, and humoral immunity and is an 'anti-inflammatory' macrophage.^40^, ^41^

Macrophages are involved in the development of psoriasis. First, macrophages are increased in psoriatic lesions.^42^ They can induce excessive proliferation and abnormal differentiation of keratinocytes by secreting cytokines such as TNF-α, MIF and IL-20.^43^, ^44^, ^45^ Second, macrophages promote psoriatic angiogenesis by upregulating the expression of Vascular Endothelial Growth Factor (VEGF), TGF-β, Platelet-Derived Growth Factor (PDGF), and TNF-α.^46^ In addition, macrophages also play a role in positive feedback of inflammatory factors (e.g., IL-1 and prokineticin 2) in psoriasis.^47^

PD-1 has been found to be expressed on macrophages and mediates macrophage apoptosis.^48^, ^49^, ^50^ In the treatment of tumors, the use of PD-1 inhibitors can attenuate this effect and increase the number of macrophages. In addition, Tumor-Associated Macrophages (TAMs) have plasticity and can differentiate into the inflammatory M1 type or the M2 type.^51^, ^52^, ^53^ PD-1+ TAMs have similar surface molecular expression patterns and functions to M2-type macrophages, such as persistently enhanced expression of CD206 and IL-10, reduced expression of HLA-DR, CD64 and IL-12, and significantly enhanced ability to suppress CD8 + T-cells.^54^ In contrast, the expression profile of PD-1-TAMs was similar to that of M1-type macrophages. Their number and the anti-inflammatory function of PD-1+ TAMs were suppressed after the use of PD-1 inhibitors. However, whether the above is involved in the development or exacerbation of psoriasis needs to be verified by relevant experiments.

### Effects of PD-1 inhibitors on the adaptive immune system

#### Potential effects of PD-1 inhibitors on Th1 and Th17 cells

Classical psoriasis is generally considered as a Th1 and Th17 disease. It has been demonstrated that in plaque psoriasis, cytokines of the Th1 pathway (IL-2, IL-12, IF-γ) and Th17A pathway (IL-17A, IL-17F, IL-21, IL-22) play a major role in the recruitment of more immune cells, leading to persistent inflammatory infiltration and proliferation of keratinocytes.^55^, ^56^ T-cells commonly express PD-1 and PD-L1, and treatment with anti-PD-1 antibodies has a dramatic effect on T-cell-mediated adaptive immunogenesis. This is often considered to be the main cause of psoriasis onset or exacerbation by anti-PD-1 antibodies. Below the authors analyze it from the perspectives of proliferation, differentiation, and function of T-cells.

In terms of proliferation, Lebwohl et al. suggested that reduced PD-L1 expression in the psoriatic epidermis may allow continuous activation of T cells. This was confirmed by immunohistochemical staining, where PD-L1 expression levels were reduced on average in the psoriatic epidermis for both mRNA and protein.^57^, ^58^, ^59^, ^60^, ^61^ However, there are reports of “clonal shrinkage” after anti-PD-1 treatment, i.e. T-cell activation clones do not appear or even disappear, the mechanisms of which need to be further investigated.^62^ It is currently known that in a culture system in which PHA (phytohaemagglutinin) stimulates T-cell proliferation, PD-L1 blocks T-cell proliferation in the G0/G1 phase, down-regulates T-cell activation, inhibits T-lymphocyte responsiveness to PHA proliferation and thus reduces T-cell proliferation.

In terms of differentiation, the authors know that Th1 and Th17 cells are the pro-immune cells, while Th2 cells are the suppressor cells of psoriatic inflammation. Blocking the PD-1/PD-L pathway not only promotes T-cell proliferation but also induces the differentiation of CD4+ T-cells into Th1 and Th17 cells.^63^, ^64^ After PD-1 blocking, T-cells have been demonstrated to exhibit multiple differentiation trajectories. Among them, there is an important differentiation trajectory, which is the trajectory of naïve T-cells → Th1 cells, or Tfh cells.^65^ This may have important implications for exploring the mechanism of psoriasis *de novo* or exacerbation by PD-1 checkpoint inhibitors.

In terms of function, the effector properties of CD4+ and CD8+ T-cells are enhanced after PD-L1 blockade treatment. Research has found that after anti-PD-L1 treatment, T-cells chemotactic to CXCL13 exhibit enhanced cytotoxicity, as well as enhancement of IFN-γ signal pathways.^66^

#### Potential effect of PD-1 inhibitors on regulatory T cells

The effect of PD1/PD-L1 on the regulation of regulatory T-cells (Treg) is mainly reflected in the following aspects. First, it has been shown that PD-L1-coated microbeads can induce Treg in vitro, and PD-L1 can increase Foxp 3 expression and enhance the immunosuppressive capacity of Treg. ^3^ In addition, PD-Ll can promote the conversion of naïve CD 4+ T-cells into Treg by downregulating Akt, mTOR and ERK 2 as well as upregulating PTEN at the same time.^67^ Second, the regulation of PD-1 is affected by the Notch pathway, and the differentiation and function of Treg also require the participation of the Notch pathway.^68^, ^69^, ^70^ Overexpression of Jagl, a ligand of the Notch pathway, on DCs, increases PD-Ll expression, and co-culture of CD 4+ T-cells with these Jagl-DCs promotes Treg generation, while PD-Ll blockade partially reduces Treg amplification.^71^ The above findings confirm the important role of PD-1 in the induction of Treg amplification.

Furthermore, the immunomodulatory capacity of Treg is reduced in ICI-induced pneumonia, and it is speculated that diminished immunosuppression of Treg may induce a more intense Th1 immune response in ICI-induced pneumonia, and it can be ventured to speculate that this response may also play a role in the formation of psoriatic lesion plaques.^72^

In a word, antagonizing PD1/PD-L1 can weaken the quantity and function of Treg, possibly leading to an imbalance between Treg and Th1/Th17 cells.^73^, ^74^, ^75^, ^76^ Therefore, it can theoretically lead to the occurrence or exacerbation of psoriasis.

#### The potential effect of PD-1 inhibitor on tissue-resident memory T-cells

Memory T-cells, based on their migratory behavior, are divided into circulating memory T-cells and Tissue-Resident Memory T-cells (TRMs).^77^ TRMs generally do not re-enter the bloodstream and persist in peripheral tissues such as epithelial barriers (the skin, the lung, the gastrointestinal tract, and the reproductive tract) and internal organs (kidney, brain), where they can provide long-term immune protection against infection, tumor, and other tissue damage. TRMs are characterized by adhesion molecule CD103 (aE integrin subunit) and/or activation marker CD69.^78^, ^79^, ^80^ TRMs have prominent characteristics of long lifespan and low mobility, as well as unique transcriptional profiles. They are usually believed to be involved in the recurrence of psoriasis.^81^, ^82^, ^83^

**Figure 1:**
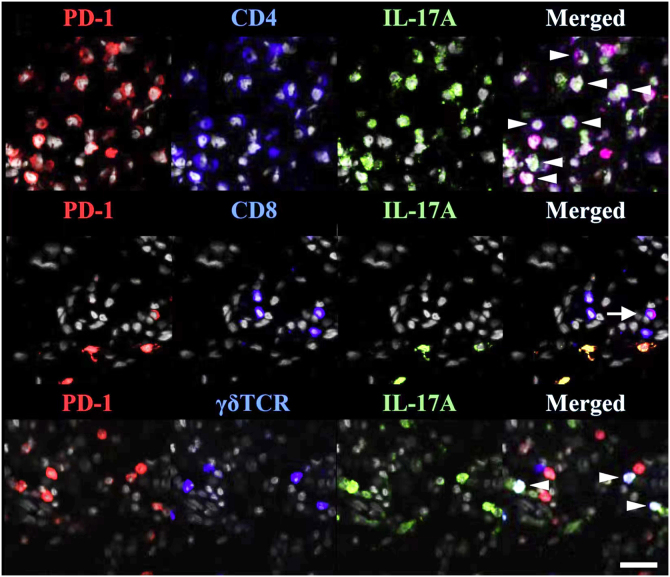
TRMs express PD-1 in psoriasis. PD-1 and IL-17A were costained with CD4, CD8, or TCR γδ. White arrowheads indicate triple-stained cells, and a tailed white arrow indicates IL-17A-PD-1^+^ CD8 T-cells. Reproduced with the permission of Copyright Clearance Center@Programmed cell death ligand 1 alleviates psoriatic inflammation by suppressing IL-17A production from programmed cell death 1-high T-cells.

TRMs express PD-1 and PD-L1(Fig. 1), suggesting that anti-PD-1/PD-L1 drugs can act on TRMs.^77^ The involvement of anti-PD-1 antibodies in the occurrence/exacerbation of psoriasis may be related to the following mechanisms: (i) Anti PD-1 therapy enhances the cytotoxic effect of CD103+TRMs;^84^ (ii) Anti PD-1 antibody enables the amplification of TRM clones and enhances the expression of IFN-γ and IL-17 that can initiate/exacerbate psoriasis;^47^ (iii) PD-1 antibody blockade enhances the interaction and co-activation of TRM with circulating memory cells.^85^

## Treatment of psoriasis induced or aggravated by PD-1 inhibitors

In 2022, the EADV Working Group put forward management requirements and basic principles: medical interventions should vary from person to person; on the premise of alleviating irAEs and improving the quality of life, the patients' targeted therapy should be continued or adjusted. A new balance should be established between irAE treatment and targeted therapy.^86^ Once psoriasis induced or aggravated by PD-1 inhibitors is diagnosed, dermatologists should be invited to participate in early treatment and management in accordance with interdisciplinary principles.^87^, ^88^

Psoriasis due to ICIs (immune checkpoint inhibitors) can be classified as Grade I, Grade II and Grade III according to the Common Criteria for the Evaluation of Adverse Events (CTCAE v5.0) classification. The majority of these patients are in Grade I or II, and only a few reach Grade III, which is in accordance with the “pyramid” pattern. According to the latest management standards by the EADV working group: Patients with Grade 1 should receive topical treatment modalities such as potent corticosteroids and vitamin D analogs while maintaining their current ICI dose; Grade II patients should be treated with traditional systemic therapies such as phototherapy and retinoids on the basis of Grade I management, with continuation or adjustment of ICI dose. Their grading needs to be re-evaluated two weeks later; Grade III patients should be treated with increased doses of traditional systemic therapy based on Grade II management. If ineffective or deteriorating, biologics (e.g., TNF-α antagonists) can be considered, and the ICI regimen should be adjusted at the same time.^86^ These differ markedly from the principles of management of traditional drug-induced psoriasis.

In the course of treatment, we should also pay attention to another issue. In the past, oncologists often recommended systemic corticosteroids for severe irAEs, however, the American Academy of Dermatology-National Psoriasis Foundation do not recommend it for psoriasis *de novo* or exacerbation by PD-1 checkpoint inhibitors. This is because systemic corticosteroids lack lasting efficacy and may cause psoriasis relapse when tapering.^89^ Also, due to the presence of malignant neoplastic disease, patients are advised to avoid cyclosporine and some biological agents and small molecule inhibitors (e.g., Jak inhibitors) that may induce/exacerbate tumor progression.^90^ While receiving treatments, patients should also avoid factors that may induce or aggravate psoriasis, such as stress, obesity, alcoholism, smoking and infection.

Taken together, we need to take into account both the following aspects: (1) The appearance of skin irAEs may be an indicator of good anti-tumor efficacy of ICIs, and (2) The possibility of psoriatic lesion recurrence or even worsening by continued immunotherapy;^91^, ^92^, ^93^ In addition, the authors need to take into account that the treatment regimen adopted should be effective against cutaneous irAEs, but also that the regimen should not negatively affect antitumor immunity. Therefore, physicians need to make individualized decisions and fully assess the risks and benefits.

## Summary

With the wide application of tumor immunotherapy, the occurrence of skin adverse reactions becomes more and more frequent. It has been a challenge for both oncologists and dermatologists to standardize the management of skin adverse reactions while minimizing the impact on immunotherapy. What the authors present here is an overview of the current status of psoriasis *de novo* or exacerbation by PD-1 checkpoint inhibitors. The authors can see that there are some aspects of this field that we need to further elucidate, such as what are the exact molecular mechanisms by which PD-1 inhibitors trigger/exacerbate psoriasis. Do these mechanisms differ and intersect with those of classical psoriasis? Are there differences in the clinical phenotypes and molecular mechanisms by which different PD-1 inhibitors trigger/exacerbate psoriasis? Is it appropriate that methotrexate is proposed to be used in the treatment of psoriasis associated with PD-1 inhibitors, as methotrexate may cause Methotrexate-associated Lymphoproliferative Disorder (MTX-LPD) in individuals with unstable immune microenvironment? The resolution of the above questions will better facilitate our effective and safe treatment of numerous skin irAEs by PD-1 inhibitors, including psoriasis.

## Financial support

National Natural Science Foundation of China (nº 82273537).

## Authors’ contributions

Zhu Shen: Conceived and designed the study, critically revised the manuscript, and approved the final version of the manuscript and the publication.

Zi Wan: Searched the literature, wrote the paper, and approved the final version of the manuscript and the publication; Approval of the final version of the manuscript; Study conception and planning; Preparation and writing of the manuscript; Manuscript critical review.

Jiangyuan Huang: Searched the literature, wrote the paper, and wrote the paper and approved the final version of the manuscript and the publication; Approval of the final version of the manuscript; Study conception and planning; Preparation and writing of the manuscript; Manuscript critical review.

Xiaojie Ou: Searched the literature and wrote the paper and wrote the paper and approved the final version of the manuscript and the publication; Approval of the final version of the manuscript; Study conception and planning; Preparation and writing of the manuscript; Manuscript critical review.

Shuang Lou: Searched the literature, wrote the paper, and approved the final version of the manuscript and the publication.

Jianji Wan: Searched the literature, critically revised the manuscript, and approved the final version of the manuscript and the publication.

## Conflicts of interest

None declared.

## References

[1] Ainsworth C. (2012). Immunology: a many layered thing. Nature.

[2] Fife B.T., Pauken K.E. (2011). The role of the PD-1 pathway in autoimmunity and peripheral tolerance. Ann N Y Acad Sci.

[3] Francisco L.M., Sage P.T., Sharpe A.H. (2010). The PD-1 pathway in tolerance and autoimmunity. Immunol Rev.

[4] Zhang X., Huang Y., Yang X. (2021). The complex role of PD-L1 in antitumor immunity: a recent update. Cell Mol Immunol.

[5] Brahmer J.R., Drake C.G., Wollner I., Powderly J.D., Picus J., Sharfman W.H. (2010). Phase I study of single-agent anti-programmed death-1 (MDX-1106) in refractory solid tumors: safety, clinical activity, pharmacodynamics, and immunologic correlates. J Clin Oncol.

[6] Sunshine J., Taube J.M. (2015). PD-1/PD-L1 inhibitors. Curr Opin Pharmacol.

[7] Nagai H., Muto M. (2018). Optimal management of immune-related adverse events resulting from treatment with immune checkpoint inhibitors: a review and update. Int J Clin Oncol.

[8] Buchbinder E.I., Desai A. (2016). CTLA-4 and PD-1 pathways: similarities, differences, and implications of their inhibition. Am J Clin Oncol.

[9] Hawkes E.A., Grigg A., Chong G. (2015). Programmed cell death-1 inhibition in lymphoma. Lancet Oncol.

[10] Weber J.S., Postow M., Lao C.D., Schadendorf D. (2016). Management of adverse events following treatment with anti-programmed death-1 agents. Oncologist.

[11] Delaunay M., Cadranel J., Lusque A., Meyer N., Gounant V., Moro-Sibilot D. (2017). Immune-checkpoint inhibitors associated with interstitial lung disease in cancer patients. Eur Respir J.

[12] Soularue E., Lepage P., Colombel J.F., Coutzac C., Faleck D., Marthey L. (2018). Enterocolitis due to immune checkpoint inhibitors: a systematic review. Gut.

[13] Dougan M., Blidner A.G., Choi J., Cooksley T., Glezerman I., Ginex P. (2020). Multinational association of supportive care in cancer (MASCC) 2020 clinical practice recommendations for the management of severe gastrointestinal and hepatic toxicities from checkpoint inhibitors. Support Care Cancer.

[14] Barroso-Sousa R., Barry W.T., Garrido-Castro A.C., Hodi F.S., Min L., Krop I.E. (2018). Incidence of endocrine dysfunction following the use of Different immune checkpoint inhibitor regimens: a systematic review and meta-analysis. JAMA Oncol.

[15] Spiers L., Coupe N., Payne M. (2019). Toxicities associated with checkpoint inhibitors-an overview. Rheumatology (Oxford).

[16] Jfri A., Leung B., Said J.T., Semenov Y., LeBoeuf N.R. (2022). Prevalence of inverse psoriasis subtype with immune checkpoint inhibitors. Immunother Adv.

[17] Vastarella M., Fabbrocini G., Sibaud V. (2020). Hyperkeratotic skin adverse events induced by anticancer treatments: a comprehensive review. Drug Saf.

[18] Andrade M.M.D.L., Tejada G.L., Peruzzo J., Bonamigo R.R. (2024). Pustular psoriasis triggered by therapy with atezolizumab and bevacizumab. An Bras Dermatol.

[19] Said J.T., Elman S.A., Perez-Chada L.M., Mita C., Merola J.F., LeBoeuf N.R. (2022). Treatment of immune checkpoint inhibitor-mediated psoriasis: a systematic review. J Am Acad Dermatol.

[20] Nikolaou V., Sibaud V., Fattore D., Sollena P., Ortiz-Brugues A., Giacchero D. (2021). Immune checkpoint-mediated psoriasis: a multicenter European study of 115 patients from the European network for cutaneous adverse event to oncologic drugs (ENCADO) group. J Am Acad Dermatol.

[21] Weber J.S., Hodi F.S., Wolchok J.D., Topalian S.L., Schadendorf D., Larkin J. (2017). Safety profile of nivolumab monotherapy: a pooled analysis of patients with advanced melanoma. J Clin Oncol.

[22] De Bock M., Hulstaert E., Kruse V., Brochez L. (2018). Psoriasis vulgaris exacerbation during treatment with a PD-1 checkpoint inhibitor: case report and literature review. Case Rep Dermatol.

[23] Kato Y., Otsuka A., Miyachi Y., Kabashima K. (2016). Exacerbation of psoriasis vulgaris during nivolumab for oral mucosal melanoma. J Eur Acad Dermatol Venereol.

[24] Ohtsuka M., Miura T., Mori T., Ishikawa M., Yamamoto T. (2015). Occurrence of psoriasiform eruption during nivolumab therapy for primary oral mucosal melanoma. JAMA Dermatol.

[25] Balak D.M., Hajdarbegovic E. (2017). Drug-induced psoriasis: clinical perspectives. Psoriasis (Auckl).

[26] Justiniano H., Berlingeri-Ramos A.C., Sanchez J.L. (2008). Pattern analysis of drug-induced skin diseases. Am J Dermatopathol.

[27] Toi Y., Sugawara S., Kawashima Y., Aiba T., Kawana S., Saito R. (2018). Association of immune-related adverse events with clinical benefit in patients with advanced non-small-cell lung cancer treated with nivolumab. Oncologist.

[28] Rogado J., Sanchez-Torres J.M., Romero-Laorden N., Ballesteros A.I., Pacheco-Barcia V., Ramos-Levi A. (2019). Immune-related adverse events predict the therapeutic efficacy of anti-PD-1 antibodies in cancer patients. Eur J Cancer.

[29] Tang K., Seo J., Tiu B.C., Le T.K., Pahalyants V., Raval N.S. (2022). Association of cutaneous immune-related adverse events with increased survival in patients treated with anti-programmed cell death 1 and anti-programmed cell death ligand 1 therapy. JAMA Dermatol.

[30] Boehncke W.H., Schon M.P. (2015). Psoriasis. Lancet.

[31] Glowacka E., Lewkowicz P., Rotsztejn H., Zalewska A. (2010). IL-8, IL-12 and IL-10 cytokines generation by neutrophils, fibroblasts and neutrophils- fibroblasts interaction in psoriasis. Adv Med Sci.

[32] Wang J.F., Wang Y.P., Xie J., Zhao Z.Z., Gupta S., Guo Y. (2021). Upregulated PD-L1 delays human neutrophil apoptosis and promotes lung injury in an experimental mouse model of sepsis. Blood.

[33] El-Benna J., Dang P.M. (2021). Live or die: PD-L1 delays neutrophil apoptosis. Blood.

[34] Kamata M., Tada Y. (2022). Dendritic cells and macrophages in the pathogenesis of psoriasis. Front Immunol.

[35] Curiel T.J., Wei S., Dong H., Alvarez X., Cheng P., Mottram P. (2003). Blockade of B7-H1 improves myeloid dendritic cell-mediated antitumor immunity. Nat Med.

[36] Zou W., Chen L. (2008). Inhibitory B7-family molecules in the tumour microenvironment. Nat Rev Immunol.

[37] Takemoto N., Intlekofer A.M., Northrup J.T., Wherry E.J., Reiner S.L. (2006). Cutting edge: IL-12 inversely regulates T-bet and eomesodermin expression during pathogen-induced CD8+ T cell differentiation. J Immunol.

[38] Zhou Y., Xu F., Chen X.Y., Yan B.X., Wang Z.Y., Chen S.Q. (2022). The epidermal immune microenvironment plays a dominant role in psoriasis development, as revealed by mass cytometry. Cell Mol Immunol.

[39] Pollard JW (2009). Trophic macrophages in development and disease. Nat Rev Immunol.

[40] Gordon S. (2003). Alternative activation of macrophages. Nat Rev Immunol.

[41] Ricardo S.D., van Goor H., Eddy A.A. (2008). Macrophage diversity in renal injury and repair. J Clin Invest.

[42] Wang H., Peters T., Sindrilaru A., Scharffetter-Kochanek K. (2009). Key role of macrophages in the pathogenesis of CD18 hypomorphic murine model of psoriasis. J Invest Dermatol.

[43] Sa S.M., Valdez P.A., Wu J., Jung K., Zhong F., Hall L. (2007). The effects of IL-20 subfamily cytokines on reconstituted human epidermis suggest potential roles in cutaneous innate defense and pathogenic adaptive immunity in psoriasis. J Immunol.

[44] Fonseca-Camarillo G., Furuzawa-Carballeda J., Llorente L., Yamamoto-Furusho J.K. (2013). IL-10-- and IL-20--expressing epithelial and inflammatory cells are increased in patients with ulcerative colitis. J Clin Immunol.

[45] Gesser B., Rasmussen M.K., Raaby L., Rosada C., Johansen C., Kjellerup R.B. (2011). Dimethylfumarate inhibits MIF-induced proliferation of keratinocytes by inhibiting MSK1 and RSK1 activation and by inducing nuclear p-c-Jun (S63) and p-p53 (S15) expression. Inflamm Res.

[46] Liu Y., Yang G., Zhang J., Xing K., Dai L., Cheng L. (2015). Anti-TNF-alpha monoclonal antibody reverses psoriasis through dual inhibition of inflammation and angiogenesis. Int Immunopharmacol.

[47] He X., Shen C., Lu Q., Li J., Wei Y., He L. (2016). Prokineticin 2 plays a pivotal role in psoriasis. EBioMedicine.

[48] Bally A.P., Lu P., Tang Y., Austin J.W., Scharer C.D., Ahmed R. (2015). NF-kappaB regulates PD-1 expression in macrophages. J Immunol.

[49] Huang X., Venet F., Wang Y.L., Lepape A., Yuan Z., Chen Y. (2009). PD-1 expression by macrophages plays a pathologic role in altering microbial clearance and the innate inflammatory response to sepsis. Proc Natl Acad Sci U S A.

[50] Roy S., Gupta P., Palit S., Basu M., Ukil A., Das P.K. (2017). The role of PD-1 in regulation of macrophage apoptosis and its subversion by Leishmania donovani. Clin Transl Immunol.

[51] Mantovani A., Locati M. (2013). Tumor-associated macrophages as a paradigm of macrophage plasticity, diversity, and polarization: lessons and open questions. Arterioscler Thromb Vasc Biol.

[52] Qian B.Z., Pollard J.W (2010). Macrophage diversity enhances tumor progression and metastasis. Cell.

[53] Mantovani A., Sozzani S., Locati M., Allavena P., Sica A. (2002). Macrophage polarization: tumor-associated macrophages as a paradigm for polarized M2 mononuclear phagocytes. Trends Immunol.

[54] Tian X., Shen H., Li Z., Wang T., Wang S. (2019). Tumor-derived exosomes, myeloid-derived suppressor cells, and tumor microenvironment. J Hematol Oncol..

[55] Monteleone G., Pallone F., MacDonald T.T., Chimenti S., Costanzo A. (2011). Psoriasis: from pathogenesis to novel therapeutic approaches. Clin Sci (Lond).

[56] Chong H.T., Kopecki Z., Cowin A.J. (2013). Lifting the silver flakes: the pathogenesis and management of chronic plaque psoriasis. Biomed Res Int.

[57] Dong H., Zhu G., Tamada K., Chen L. (1999). B7-H1, a third member of the B7 family, co-stimulates T-cell proliferation and interleukin-10 secretion. Nat Med.

[58] Latchman Y., Wood C.R., Chernova T., Chaudhary D., Borde M., Chernova I. (2001). PD-L2 is a second ligand for PD-1 and inhibits T cell activation. Nat Immunol.

[59] Yokosuka T., Takamatsu M., Kobayashi-Imanishi W., Hashimoto-Tane A., Azuma M., Saito T. (2012). Programmed cell death 1 forms negative costimulatory microclusters that directly inhibit T cell receptor signaling by recruiting phosphatase SHP2. J Exp Med.

[60] Carter L., Fouser L.A., Jussif J., Fitz L., Deng B., Wood C.R. (2002). PD-1:PD-L inhibitory pathway affects both CD4(+) and CD8(+) T cells and is overcome by IL-2. Eur J Immunol.

[61] Wang T.T., Zhao Y.L., Peng L.S., Chen N., Chen W., Lv Y.P. (2017). Tumour-activated neutrophils in gastric cancer foster immune suppression and disease progression through GM-CSF-PD-L1 pathway. Gut.

[62] Bassez A., Vos H., Van Dyck L., Floris G., Arijs I., Desmedt C. (2021). A single-cell map of intratumoral changes during anti-PD1 treatment of patients with breast cancer. Nat Med.

[63] Iwai Y., Ishida M., Tanaka Y., Okazaki T., Honjo T., Minato N. (2002). Involvement of PD-L1 on tumor cells in the escape from host immune system and tumor immunotherapy by PD-L1 blockade. Proc Natl Acad Sci U S A.

[64] Dulos J., Carven G.J., van Boxtel S.J., Evers S., Driessen-Engels L.J., Hobo W. (2012). PD-1 blockade augments Th1 and Th17 and suppresses Th2 responses in peripheral blood from patients with prostate and advanced melanoma cancer. J Immunother.

[65] Street K., Risso D., Fletcher R.B., Das D., Ngai J., Yosef N. (2018). Slingshot: cell lineage and pseudotime inference for single-cell transcriptomics. BMC Genomics.

[66] Zhang Y., Chen H., Mo H., Hu X., Gao R., Zhao Y. (2021). Single-cell analyses reveal key immune cell subsets associated with response to PD-L1 blockade in triple-negative breast cancer. Cancer Cell.

[67] Francisco L.M., Salinas V.H., Brown K.E., Vanguri V.K., Freeman G.J., Kuchroo V.K. (2009). PD-L1 regulates the development, maintenance, and function of induced regulatory T cells. J Exp Med.

[68] Mathieu M., Cotta-Grand N., Daudelin J.F., Thebault P., Labrecque N. (2013). Notch signaling regulates PD-1 expression during CD8(+) T-cell activation. Immunol Cell Biol.

[69] Pan T., Liu Z., Yin J., Zhou T., Liu J., Qu H. (2015). Notch signaling pathway was involved in regulating programmed cell death 1 expression during sepsis-induced immunosuppression. Mediators Inflamm.

[70] Tindemans I., Peeters M.J.W., Hendriks R.W. (2017). Notch signaling in T Helper Cell subsets: instructor or unbiased amplifier?. Front Immunol.

[71] Lin C.L., Huang H.M., Hsieh C.L., Fan C.K., Lee Y.L. (2019). Jagged1-expressing adenovirus-infected dendritic cells induce expansion of Foxp3(+) regulatory T cells and alleviate T helper type 2-mediated allergic asthma in mice. Immunology.

[72] Franken A., Van Mol P., Vanmassenhove S., Donders E., Schepers R., Van Brussel T. (2022). Single-cell transcriptomics identifies pathogenic T-helper 17.1 cells and pro-inflammatory monocytes in immune checkpoint inhibitor-related pneumonitis. J Immunother Cancer.

[73] Harrington L.E., Hatton R.D., Mangan P.R., Turner H., Murphy T.L., Murphy K.M. (2005). Interleukin 17-producing CD4+ effector T cells develop via a lineage distinct from the T helper type 1 and 2 lineages. Nat Immunol.

[74] Park H., Li Z., Yang X.O., Chang S.H., Nurieva R., Wang Y.H. (2005). A distinct lineage of CD4 T cells regulates tissue inflammation by producing interleukin 17. Nat Immunol..

[75] Zhang L., Li Y., Yang X., Wei J., Zhou S., Zhao Z. (2016). Characterization of Th17 and FoxP3(+) treg cells in paediatric psoriasis patients. Scand J Immunol.

[76] Soler D.C., Sugiyama H., Young A.B., Massari J.V., McCormick T.S., Cooper K.D. (2013). Psoriasis patients exhibit impairment of the high potency CCR5(+) T regulatory cell subset. Clin Immunol.

[77] Sallusto F., Lenig D., Forster R., Lipp M., Lanzavecchia A. (1999). Two subsets of memory T lymphocytes with distinct homing potentials and effector functions. Nature.

[78] Masopust D., Vezys V., Marzo A.L., Lefrancois L. (2001). Preferential localization of effector memory cells in nonlymphoid tissue. Science.

[79] Reinhardt R.L., Khoruts A., Merica R., Zell T., Jenkins M.K. (2001). Visualizing the generation of memory CD4 T cells in the whole body. Nature.

[80] Mackay L.K., Rahimpour A., Ma J.Z., Collins N., Stock A.T., Hafon M.L. (2013). The developmental pathway for CD103(+)CD8+ tissue-resident memory T cells of skin. Nat Immunol.

[81] Tokura Y., Phadungsaksawasdi P., Kurihara K., Fujiyama T., Honda T. (2021). Pathophysiology of skin resident memory T Cells. Front Immunol.

[82] Crowl J.T., Heeg M., Ferry A., Milner J.J., Omilusik K.D., Toma C. (2022). Tissue-resident memory CD8(+) T cells possess unique transcriptional, epigenetic and functional adaptations to different tissue environments. Nat Immunol.

[83] Zaid A., Mackay L.K., Rahimpour A., Braun A., Veldhoen M., Carbone F.R. (2014). Persistence of skin-resident memory T cells within an epidermal niche. Proc Natl Acad Sci U S A.

[84] Banchereau R., Chitre A.S., Scherl A., Wu T.D., Patil N.S., de Almeida P. (2021). Intratumoral CD103+ CD8+ T cells predict response to PD-L1 blockade. J Immunother Cancer.

[85] Ridge J.P., Di Rosa F., Matzinger P. (1998). A conditioned dendritic cell can be a temporal bridge between a CD4+ T-helper and a T-killer cell. Nature.

[86] Apalla Z., Nikolaou V., Fattore D., Fabbrocini G., Freites-Martinez A., Sollena P. (2022). European recommendations for management of immune checkpoint inhibitors-derived dermatologic adverse events. The EADV task force’ Dermatology for cancer patients’ position statement. J Eur Acad Dermatol Venereol.

[87] Tattersall I.W., Leventhal J.S. (2020). Cutaneous toxicities of immune checkpoint inhibitors: the role of the dermatologist. Yale J Biol Med.

[88] Gravalos C., Sanmartin O., Gurpide A., Espana A., Majem M., Suh Oh H.J. (2019). Clinical management of cutaneous adverse events in patients on targeted anticancer therapies and immunotherapies: a national consensus statement by the Spanish Academy of Dermatology and Venereology and the Spanish Society of Medical Oncology. Clin Transl Oncol.

[89] Menter A., Gelfand J.M., Connor C., Armstrong A.W., Cordoro K.M., Davis D.M.R. (2020). Joint American Academy of Dermatology-National Psoriasis Foundation guidelines of care for the management of psoriasis with systemic nonbiologic therapies. J Am Acad Dermatol.

[90] Dougan M., Luoma A.M., Dougan S.K., Wucherpfennig K.W. (2021). Understanding and treating the inflammatory adverse events of cancer immunotherapy. Cell.

[91] Chan L., Hwang S.J.E., Byth K., Kyaw M., Carlino M.S., Chou S. (2020). Survival and prognosis of individuals receiving programmed cell death 1 inhibitor with and without immunologic cutaneous adverse events. J Am Acad Dermatol.

[92] Aso M., Toi Y., Sugisaka J., Aiba T., Kawana S., Saito R. (2020). Association between skin reaction and clinical benefit in patients treated with anti-programmed cell death 1 monotherapy for advanced non-small cell lung cancer. Oncologist.

[93] Simonaggio A., Michot J.M., Voisin A.L., Le Pavec J., Collins M., Lallart A. (2019). Evaluation of readministration of immune checkpoint inhibitors after immune-related adverse events in patients with cancer. JAMA Oncol.

